# Functional Role of Cyclin-Dependent Kinase 5 in the Regulation of Melanogenesis and Epidermal Structure

**DOI:** 10.1038/s41598-017-12567-1

**Published:** 2017-10-23

**Authors:** Changsheng Dong, Shanshan Yang, Ruiwen Fan, Kaiyuan Ji, Junzhen Zhang, Xuexian Liu, Shuaipeng Hu, Jianshan Xie, Yu Liu, Wenjun Gao, Haidong Wang, Jianbo Yao, George W Smith, Muren Herrid

**Affiliations:** 1 College of Animal Science and Veterinary Medicine, Shanxi Agricultural University, Taigu, 030801 China; 2 Laboratory of Animal Biotechnology and Genomics, Division of Animal and Nutritional Sciences, West Virginia University, Morgantown, WV 26506 USA; 3 Laboratory of Mammalian Reproductive Biology and Genomics, Departments of Animal Science and Physiology, Michigan State University, East Lansing, MI 48824 USA; 4 Centre for Bioactive Discovery in Health and Aging, University of New England, Armidale, NSW2351 Australia

## Abstract

The mammalian integumentary system plays important roles in body homeostasis, and dysfunction of melanogenesis or epidermal development may lead to a variety of skin diseases, including melanoma. Skin pigmentation in humans and coat color in fleece-producing animals are regulated by many genes. Among them, microphthalmia-associated transcription factor (*MITF*) and paired-box 3 (*PAX3*) are at the top of the cascade and regulate activities of many important melanogenic enzymes. Here, we report for the first time that cyclin-dependent kinase 5 (Cdk5) is an essential regulator of *MITF* and *PAX3*. *Cdk5* knockdown in mice causes a lightened coat color, a polarized distribution of melanin and hyperproliferation of basal keratinocytes. Reduced expression of Keratin 10 (*K10*) resulting from *Cdk5* knockdown may be responsible for an abnormal epidermal structure. In contrast, overexpression of *Cdk5* in sheep (Ovis aries) only produces brown patches on a white background, with no other observable abnormalities. Collectively, our findings show that Cdk5 has an important functional role in the regulation of melanin production and transportation and in normal development of the integumentary system.

## Introduction

As the largest organ of the body, the integumentary system comprises the skin, hair, nails, and exocrine glands and plays multiple roles in homeostasis^1,2^. The skin serves as the first line of defense of the body and provides temperature regulation and some aspects of metabolism. Consequently, the color, thickness and secretions of the skin determine its ability to protect an individual from environmental and biological insults, such as ultraviolet radiation (UVR) and bacteria. Developmental defects and dysfunctions of the integumentary system are invariably associated with abnormal human and animal pathologies. Cutaneous melanoma, which arises from normal melanocytes or their precursors, is the most common form of skin cancer^3^, but the mechanism regulating epidermal development and skin pigmentation is still not fully understood.

Pigmentation of the skin, hair, and eyes depends primarily on the synthesis and distribution of melanin that is produced by melanocytes^2^. At the cellular level, melanocytes are functionally associated with fibroblasts and keratinocytes via dendrites to regulate the function and phenotype of the skin^3^. Melanin synthesis is restricted to melanocytes, where it plays a major role in antioxidant defense mechanisms^4–6^. In addition, it can also promote skin resistance to different types of cancer therapy, including chemotherapy or radiotherapy, in patients with metastatic melanoma^6^. Autocrine and paracrine regulation of melanin synthesis have been established^7^. For example, L-tyrosine and L-DOPA not only directly regulate melanogenesis but also indirectly mediate the process through specific receptor- or non-receptor-mediated processes^8^.

At the molecular level, more than 125 genes have been identified as being involved in the regulation of melanocyte development and melanogenesis at different pathways, which act at transcriptional, translational, and posttranslational levels^9^_._ Although protein kinase C (PKC) plays a regulatory role in melanogenesis, cAMP is the most important biochemical regulator. Melanocortin receptor type 1 (MC1R) is one of seven transmembrane G protein-coupled receptors involved in regulation of melanogenic activity. In melanocytes, it can regulate melanogenesis, cell proliferation and survival^7,8^. It is activated upon binding of MSH or ACTH peptides (products of POMC processing) as a result of intracellular cAMP accumulation^10^. Among the transcriptional regulators, microphthalmia-associated transcription factor (MITF) and paired-box 3 (PAX3) are two major regulators of these processes^7,11–14^. MITF represents a melanocyte-specific communication hub that integrates intracellular signals and transcriptional responses in regulating cell differentiation, proliferation and survival^15^. MITF and PAX3 regulate the transcription of genes for three major pigmentation enzymes, namely, tyrosinase (TYR), tyrosine related protein-1 (TYRP1), and tyrosine related protein-2 (TYRP2; dopachrome tautomerase (DCT))^16–19^. Furthermore, PAX3 regulates melanocyte specification, maintenance of the undifferentiated state, proliferation, and migration during embryonic development^20^. PAX3 directly interacts with the SRY-related HMG-box (Sox10) and can activate MITF expression in a synergistic manner^21^ while simultaneously competing with MITF for occupancy of an enhancer required for the expression of TYRP2^22^. In addition, our previous investigations have identified approximately 105 miRNAs that are possibly involved in the regulation of coat color genes^23^. A further study showed that overexpression of miR-137 in transgenic mice results in altered coat color, thereby validating the functional role of miR-137 on MITF gene expression and indicating the complexity of the regulation mechanisms for melanogenesis and coat color gene expression post-transcriptionally^24^.

Taking advantage of 22 natural coat-color variations in alpacas, we have constructed one cDNA library from white alpaca skin^25^ and two small RNA libraries from white and brown alpaca skin^23^. An analysis of the transcriptome profile of alpaca skin with different hair colors has revealed that cyclin-dependent kinase 5 (*Cdk5*) is a candidate gene for alpaca fleece quality, coat color and fiber growth and development^25^. Indeed, a subsequent study showed the localization of the Cdk5 protein to hair follicles, with higher expression levels in animals with a brown versus white fleece color^26^.

Cdks are serine/threonine kinases that are activated by binding to regulatory cyclins that play a key regulatory role in cell cycle progression. However, Cdk5 is unique and does not function as a cell cycle regulator. Cdk5 is best known for its role in post-mitotic processes and functions in the developing nervous system from the neural crest (NC)^27,28^. During neurulation in early development, NC stem cells arise from the dorsal neural tube and migrate throughout the body, where they give rise to critical components of numerous cell types, including melanocytes^29^. In melanocyte development, MITF is extensively involved and provides a central link between transcription factors and signaling pathways^29^. Despite the ubiquitous distribution of Cdk5 and its substrates^27,30^, Cdk5 activity has been mainly detected in the nervous system, where it is most abundantly expressed^30,31^. However, several recent studies have suggested that Cdk5 also plays a functional role outside of the nervous system, such as in muscle cells^32^, the lymphatic system^33^, the kidney^34^ and gonadal cells^35^. A functional role for Cdk5 in melanogenesis and epidermal development is still lacking.

In the present study, we first investigated the mechanism of action of Cdk5 in melanogenesis by determining the effect of Cdk5 on the expression levels of key enzymes in the melanogenesis pathway in cultured alpaca melanocytes. We then generated *Cdk5* knockdown mice and Cdk5-overexpressing sheep to further confirm the functional role of Cdk5 on melanogenesis and epidermal development *in vivo*.

## Results

### The influence of up- and down-regulation of Cdk5 on the expression levels of key enzymes in the melanogenesis pathway

**Figure 1:**
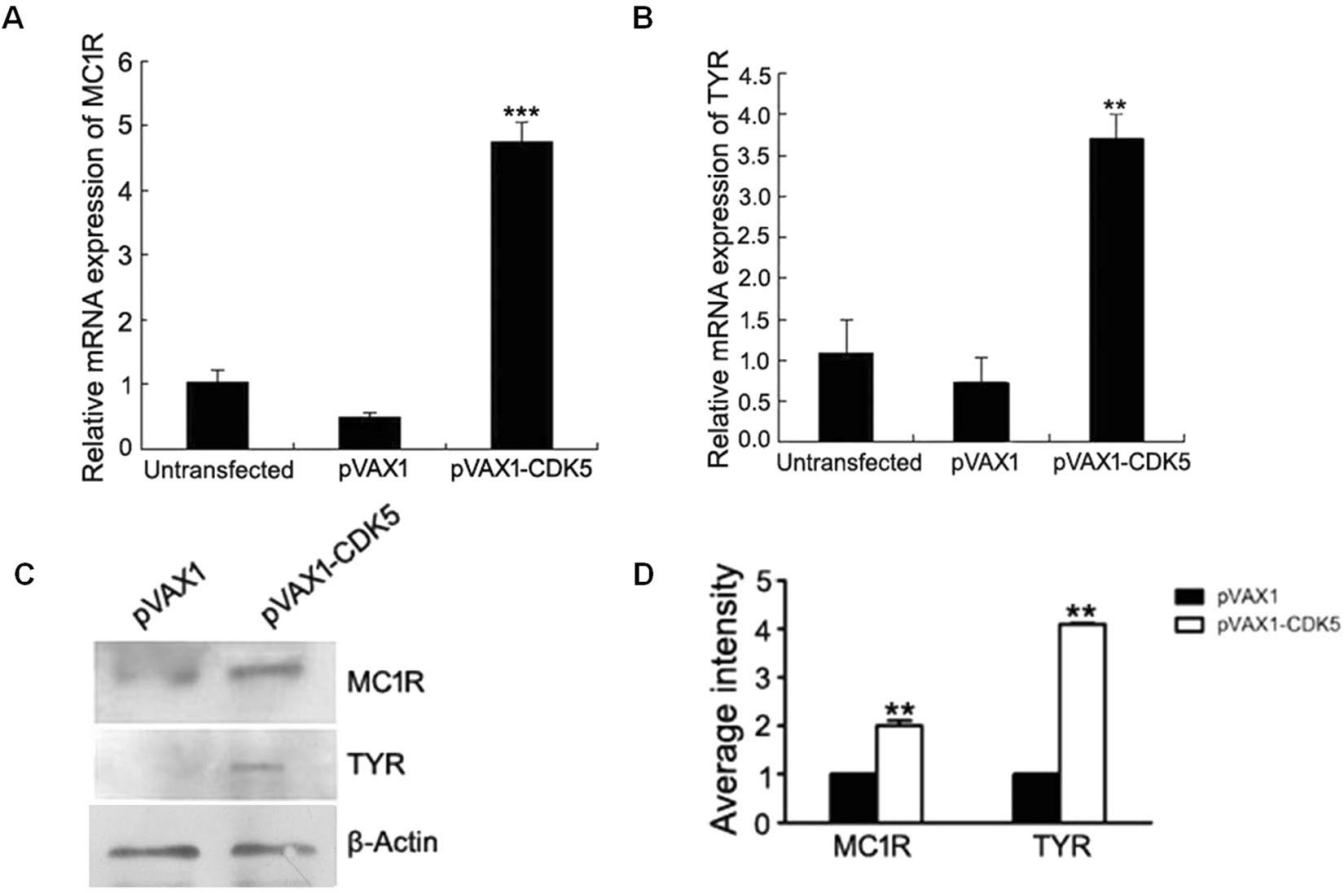
Effect of *Cdk5* overexpression on the abundance of mRNA for genes involved in coat color regulation. RT-qPCR was performed to compare expression of the MC1R (**A**) and TYR (**B**) mRNAs in melanocytes transfected with pVAX1- Cdk5 to expression in cells transfected with the pVAX1 vector. (**C**,**D**) RT-qPCR was performed to compare the protein expression of TYR and MC1R in melanocytes transfected with pVAX1- Cdk5 to expression in control cells transfected with the pVAX1 vector. The abundance of the *TYR* and *MC1R* mRNA and protein was normalized relative to the abundance of 18 S rRNA and β-actin, respectively. Bars in each panel represent the mean ± standard error (n = 3). **P < 0.05, ***P < 0.001.

Using alpaca melanocytes, we attempted to determine if Cdk5 controls the expression levels of MC1R and TYR in the melanogenesis pathway. Overexpression of *Cdk5* was induced in melanocytes by transfection of the pVAX1-Cdk5 vector. Induced overexpression of the Cdk5 gene in cultured melanocytes resulted in an increase in the expression level of *Cdk5* mRNA (Supplementary Figure 1A) compared to the controls. Western blot analysis showed an increased production of the Cdk5 protein (Supplementary Figure 1B). Furthermore, MC1R and TYR genes in the cells transfected with the pVAX1-Cdk5 vector were increased by approximately 5-fold and 3-fold, respectively, at the mRNA level, and approximately 4-fold and 3-fold at the protein level, respectively (Fig. 1A–D), compared to the cells transfected with the vector control and the non-transfected cells.

**Figure 2:**
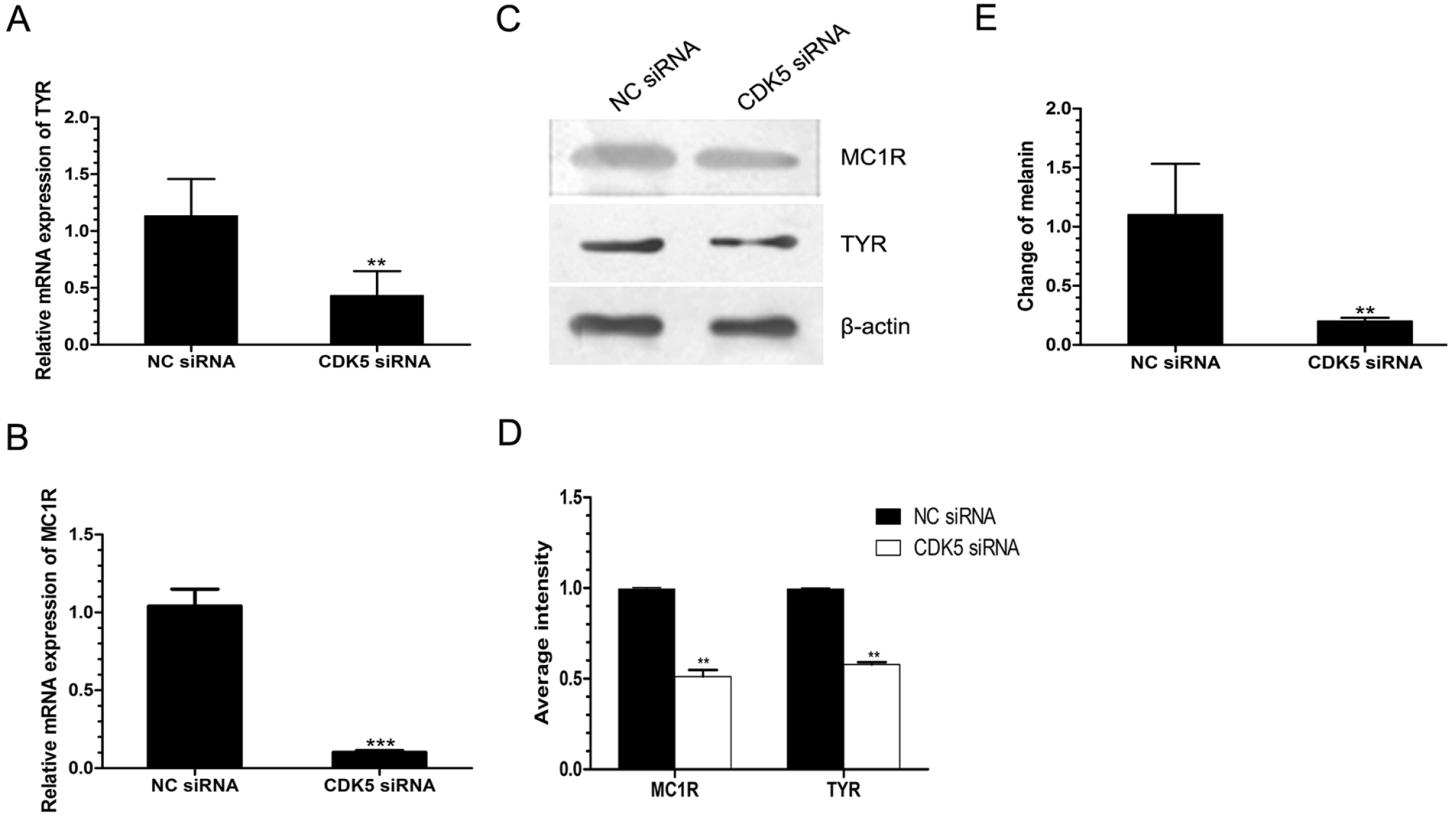
Effect of siRNA-mediated Cdk5 depletion on mRNA and protein expression for MC1R and TYR in cultured alpaca melanocytes. (**A**,**B**) RT-qPCR analysis of *TYR* and *MC1R* mRNA expression in negative control siRNA (NC)- and *Cdk5* siRNA-transfected alpaca melanocytes. (**C**,**D**) Western blot analysis of TYR and MC1R protein expression in NC siRNA- and *Cdk5* siRNA-transfected melanocytes. The abundance of the *TYR* and *MC1R* mRNA and protein was normalized relative to the abundance of 18 S rRNA and β-actin, respectively. (**E**) Spectrophotometric measurement for the total alkali-soluble melanins. Bars represent the mean ± standard error (n = 3). **P < 0.05, ***P < 0.001. The experiment was repeated three times.

To further study the effect of Cdk5 on the expression levels of key enzymes in the pathway, 3 distinct siRNAs were designed to knock down Cdk5 expression in alpaca melanocytes. Cdk5 protein expression was most dramatically decreased in melanocytes transfected with siRNA-215 compared to the other two (Supplementary Figure 1C). As shown in Supplementary Figure 1D, depletion of the *Cdk5* gene in the cells resulted in a 90% reduction in the expression level of *Cdk5* mRNA compared to the controls. Immunocytochemistry analysis showed a reduced production of the Cdk5 protein (Supplementary Figure 1E). Subsequently, the expression levels of the *MC1R* and *TYR* mRNA and protein in the siRNA-215-transfected cells were examined. In comparison to the negative controls, depletion of Cdk5 led to a significant reduction in the expression levels of the *TYR* (Fig. 2A) and *MC1R* (Fig. 2B) mRNAs and corresponding proteins (Fig. 2C and D).

Taken together, the results from the overexpression and partial depletion of Cdk5 in alpaca melanocytes suggest an important functional role for Cdk5 in the regulation of melanogenesis via the control of the MC1R and TYR genes. We next tested if the changes in the production levels of MC1R and TYR in the melanocytes influenced melanogenesis. Indeed, as shown in Fig. 2E, depletion of Cdk5 resulted in a reduction of more than 70% in the total alkali-soluble melanin in the *Cdk5* siRNA-transfected melanocytes relative to the negative controls.

**Figure 3:**
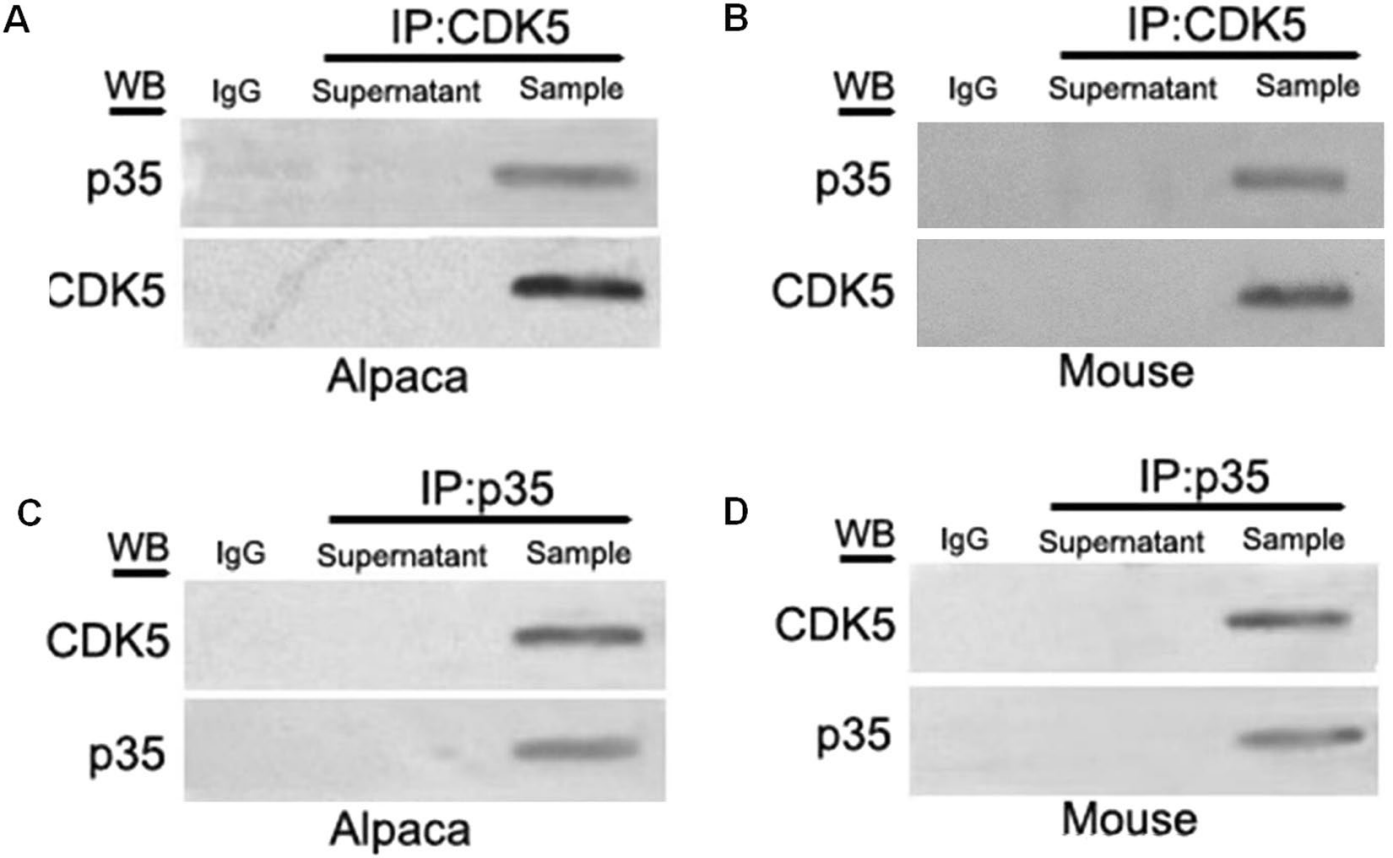
Co-immunoprecipitation (Co-IP) analysis of the interaction between Cdk5 and p35 in mouse and alpaca melanocytes. (**A**,**B**) Co-IP experiments were performed using an anti-Cdk5 antibody followed by a Western blot analysis using an anti-p35 antibody and anti-Cdk5 antibody. (**C**,**D**) Reciprocal Co-IP experiments using an anti-p35 antibody were performed followed by a Western blot analysis using an anti-Cdk5 antibody and anti-p35 antibody. IgG was used as the negative control.

Cdk5 forms a complex with its activators, p35 or p39, which have multiple functions in neurons. Notably, Cdk5/p35 plays an important role in regulating neural migration, differentiation, and synaptogenesis in immature neurons^27^. Recognizing that p35 is a prominent activator of Cdk5 in the developing cerebral cortex and hippocampus and in the embryonic origin of melanocytes from the neural crest (NC), the ability of Cdk5 to interact with p35 was examined by co-immunoprecipitation (co-IP) experiments in the melanocytes. Using anti-Cdk5 and anti-p35 antibodies, the co-IP and reciprocal co-IP experiments showed that Cdk5 interacts with p35 in the cultured melanocytes (Fig. 3).

### Phenotype of *Cdk5* knockdown mice and its possible mechanism

To investigate the role of Cdk5 in regulating melanogenesis *in vivo*, we established a knockdown mouse model. Transgenic mice were produced by microinjection of mouse zygotes with a shRNA expression vector targeting *Cdk5*. We obtained three transgenic founder animals from the transfer of 50 embryos into 6 surrogate mice. The expression of the Cdk5 protein in the skin and hair of 3 transgenic mice was decreased compared to the wild-type mice (Supplementary Figure 2), indicating the effectiveness of the *Cdk5* siRNA in reducing Cdk5 production in the skin.

**Figure 4:**
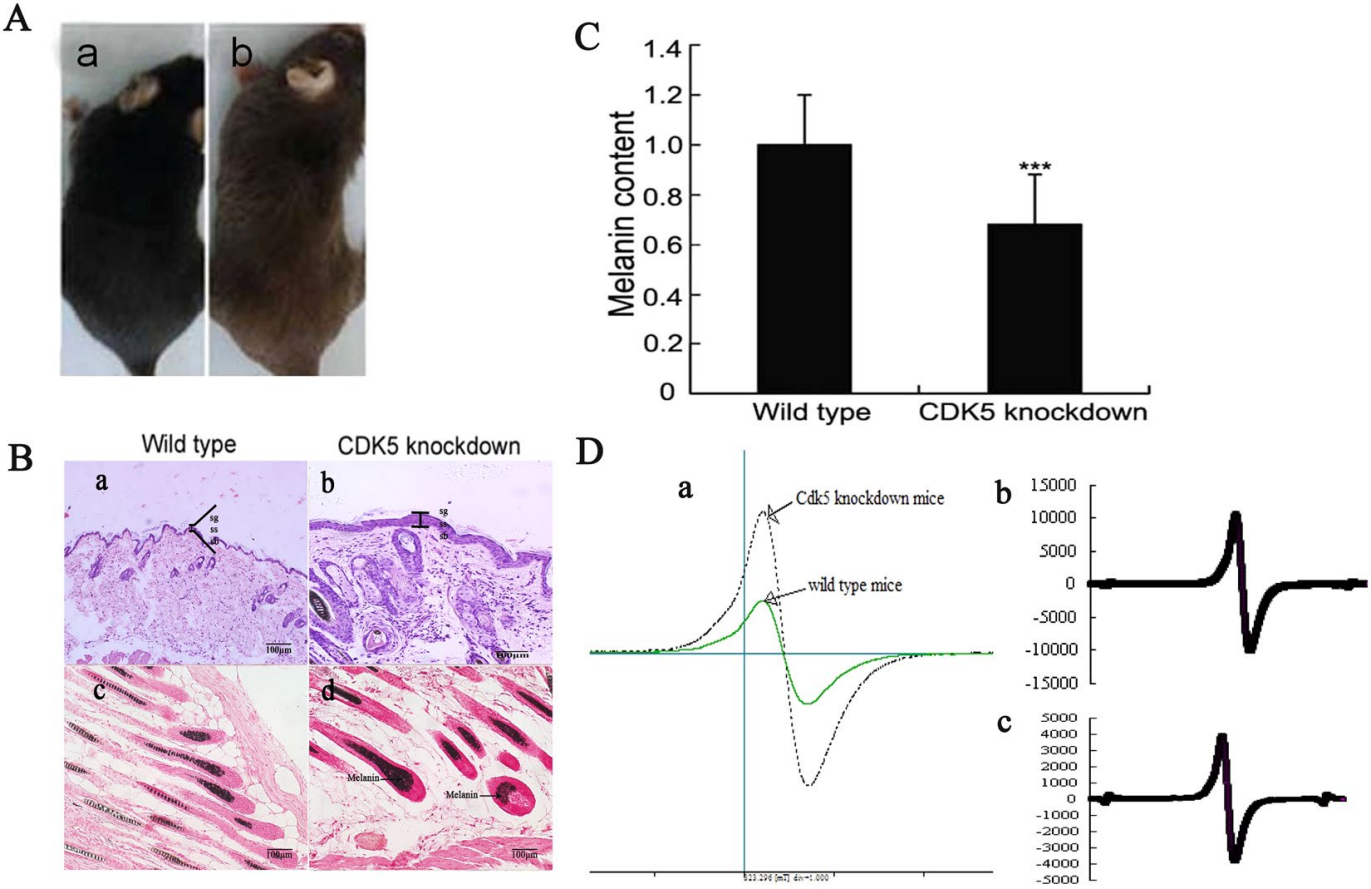
Phenotypes of *Cdk5* knockdown mice. (**A**) *Cdk5* knockdown mice showing lightened hair color compared to the wild-type mice. (**B**) (a-b) Hematoxylin staining of skin sections of *Cdk5* knockdown mice compared with the wild-type mice. sb, stratum basale; sg, stratum granulosum; ss, stratum spinosum. (c-d) Fontana-Masson staining of skin sections of *Cdk5* knockdown mice compared with the wild-type mice. (**C**) Spectrophotometric measurement for alkali-soluble melanins in the hair of *Cdk5* knockdown mice compared to the wild-type mice. Bars represent the mean ± standard error (n = 3). ***P < 0.001. (**D**) (a) comparison of the ESR signal in the hair between wild-type mice and *Cdk5* knockdown mice. b: The ESR signal of the hair in wild-type mice. (c) The ESR signal of the hair in *Cdk5* knockdown mice. The following analytical conditions were used: a microwave frequency of 9050.999 MHz, field of 323 ± 5 mT, modulation amplitude of 0.1 mT, microwave power of 0.998 mW and frequency of 9050.999 MHz, and time constant of 0.03 s. The g value was 1.99863.

The breeding of transgenic founders with wild-type mice showed no observable difference between the transgenic and wild-type mice in terms of growth rate and survival. Three litters of the F1 generation, a total of 18 viable pups, were all born at the expected time. After genotyping for the transgene by PCR, 3 mice, whose hair color was light brown (Fig. 4A), were identified as transgenic mice, and the remaining 15 non-transgenic mice were black. The three transgenic mice had a lower level of melanin than the controls (Fig. 4C). The result of the EPR signal analysis showed that the corresponding gains for a synthetic model of eumelanins in the wild-type and *Cdk5* knockdown mice, without hyperfine splitting for pheomelanins of the hair, were about 10,000 and 4,000, respectively (Fig. 4D).

**Figure 5:**
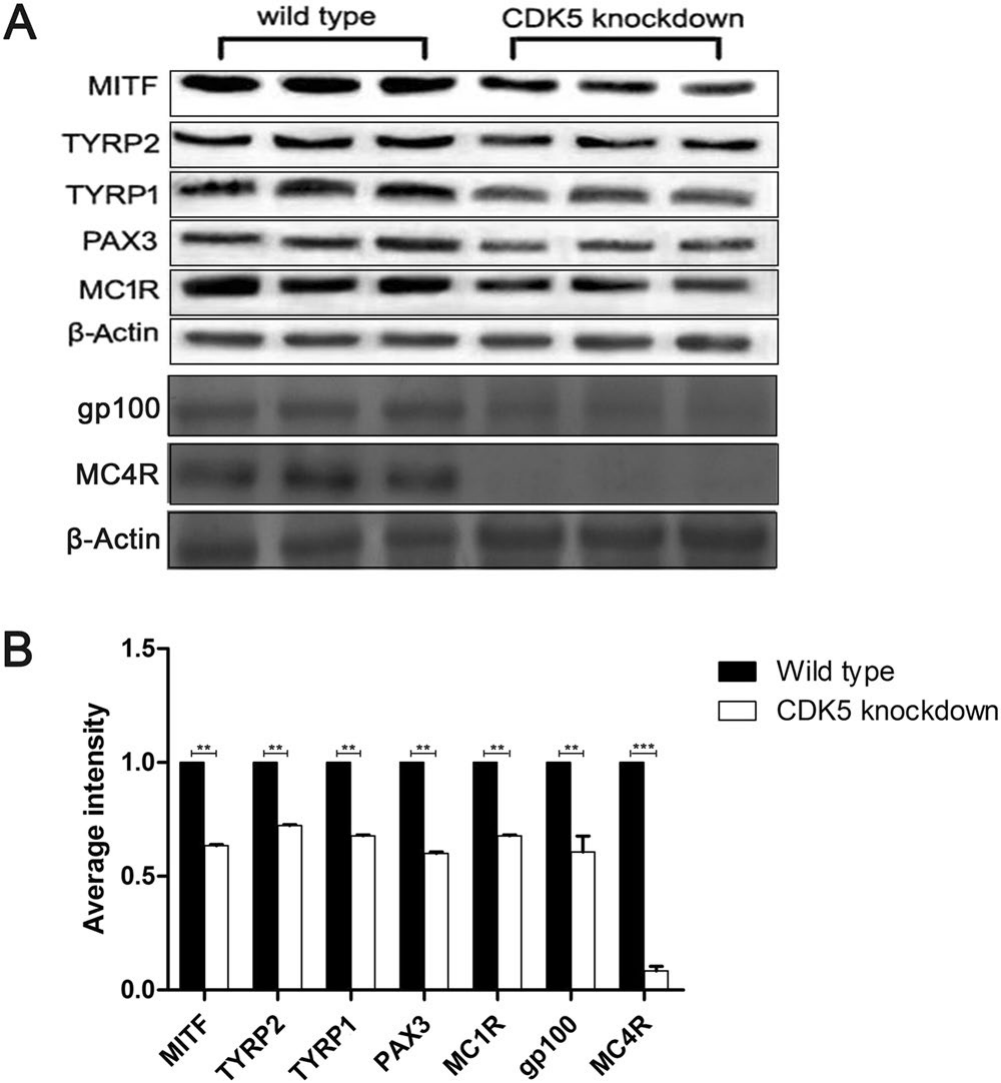
Analysis of relative hair color gene expression for evidence of a transcriptome profile, gp100 expression and MC4R protein expression in the skin of *Cdk5* knockdown mice. (**A**) Western blot analysis of MITF, TYRP1, TYRP2, PAX3, MC1R, gp100 and MC4R protein expression in the skin of *Cdk5* knockdown mice comp ompared to the wild-type mouse ared with wild-type mice. (**B**) The abundance of the MITF, TYRP1, TYRP2, PAX3 and MC1R proteins was quantified using Image-Pro Plus software and normalized relative to the abundance of β-actin. Bars represent the mean ± standard error (n = 3). ***P < 0.001.

To establish possible mechanisms by which Cdk5 regulates melanogenesis, we performed RNA-Seq and Western blot analysis of the skin from *Cdk5* knockdown mice versus wild-type mice. FDR (false discovery rate) values of ≤0.01 and an RPKM (Reads Per kb transcriptome per Million reads) ratio of >2 were used in the analysis. The expression levels of the mRNA and proteins of a number of important hair color genes, namely, *MITF, PAX3, MC1R, TYRP1*, and *TYRP2*, were down-regulated in the *Cdk5* knockdown mice (Supplementary Table 1) (Fig. 5). Western blot showed that MC4R and gp100 protein expression was down-regulated in the *Cdk5* knockdown mice (Fig. 5).

Consistent with our observation of the role of Cdk5 in regulating melanogenesis in cultured melanocytes, knockdown of *Cdk5* in mice down-regulates the expression of important hair color genes and subsequently alters the hair phenotype.

### Knockdown of *Cdk5* impairs the epidermal structure and transportation system of melanins

**Figure 6:**
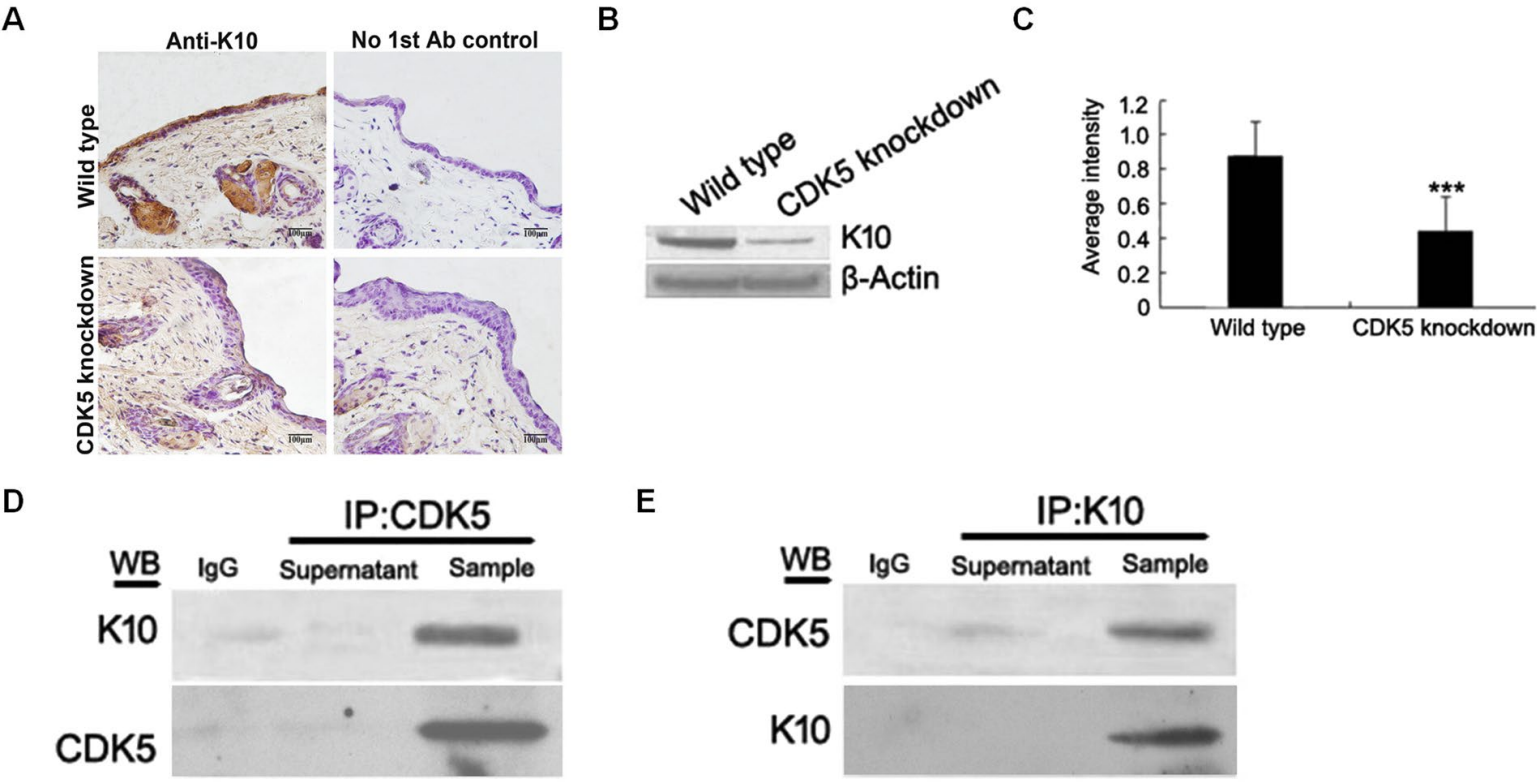
Analysis of K10 protein expression in the skin of *Cdk5* knockdown mice. (**A**) Immunohistochemical analysis of K10 protein expression in the skin of *Cdk5* knockdown mice. (**B**) Western blot analysis of K10 protein expression in the skin of *Cdk5* knockdown mice compared with wild-type mice. (**C**) The abundance of the K10 protein was quantified using Image-Pro Plus software and normalized relative to the abundance of the β-actin protein. (**D**) Co-IP experiments were performed using an anti-Cdk5 antibody followed by Western blot analysis using an anti-K10 antibody and anti-Cdk5 antibody. (**E**) Reciprocal co-IP experiments using an anti-K10 antibody were performed followed by a Western blot analysis using an anti-Cdk5 antibody and anti-K10 antibody. Bars represent the mean ± standard error (n = 3). ***P < 0.001.

At the age of 4 weeks, with the growth of visible hair, 3 transgenic mice were observed with a light brown coat color. However, at approximately 8 weeks, these animals started to lose hair and exhibited abnormal skin conditions, such as plaques of red skin. As a result, all animals were euthanized, and skin samples were collected at the age of 6 months. The skin histological analyses revealed that down-regulation of Cdk5 production in the skin caused changes in the epidermis structure. Compared to the wild-type mouse (52.11 ± 6.26 µm), the epidermis of the* Cdk5* knockdown mouse (122.57 ± 11.25 µm) was thickened, due to more stratification, especially in the stratum spinosum, and the cells of all strata were enlarged (Fig. 4B-a,b). Fontana-Masson staining showed that the melanin was localized to melanogenic melanocytes in the upper dermal papilla (Fig. 4B-c,d). To understand the mechanism responsible for the thickening of the epidermis in the *Cdk5* knockdown mice, we performed co-IPs followed by mass spectrometry to identify proteins that interacted with Cdk5 in alpaca melanocytes. Keratin 10 (K10) was identified as one of the targets that interacted with Cdk5. The interaction between the two proteins was confirmed with the co-IP experiments (Fig. 6D and E). Consequently, we tested the K10 protein levels in the skin of the *Cdk5* knockdown and wild-type mice. The Western blot results showed that the expression of the K10 protein was significantly lower in the skin of the *Cdk5* knockdown mice (P < 0.01) (Fig. 6B and C).

**Figure 7:**
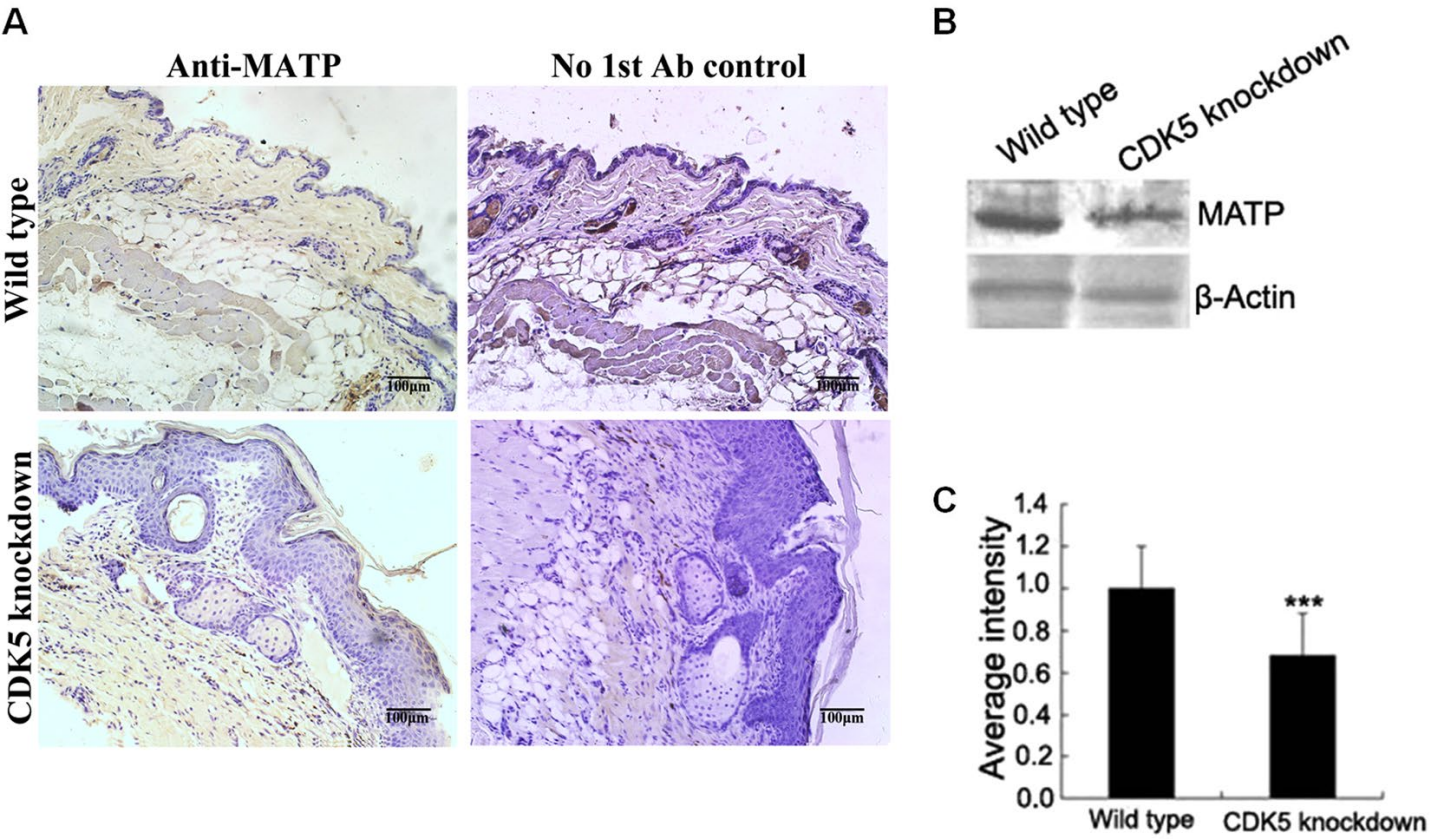
Analysis of MATP protein expression in the skin of *Cdk5* knockdown mice. (**A**) Immunohistochemical analysis of MATP protein expression in the skin of *Cdk5* knockdown mice. Scale bar: 100 μm. (**B**) Western blot analysis of MATP protein expression in the skin of *Cdk5* knockdown mice compared with wild-type mice. (**C**) The abundance of the MATP protein was quantified using Image-Pro Plus software and normalized relative to the abundance of the β-actin protein. Bars represent the mean ± standard error (n = 3). ***P < 0.001.

The polarized distribution pattern of melanin in the epidermis led to an investigation of the expression of membrane-associated transporter protein (MATP) in the skin. As shown in Fig. 7A, the density of MATP immunostaining in the skin of the knockdown mice was reduced compared to that of the wild-type mice, which was further confirmed by Western blot (Fig. 7B and C).

### Skin tissue-specific overexpression of Cdk5 in sheep

**Figure 8:**
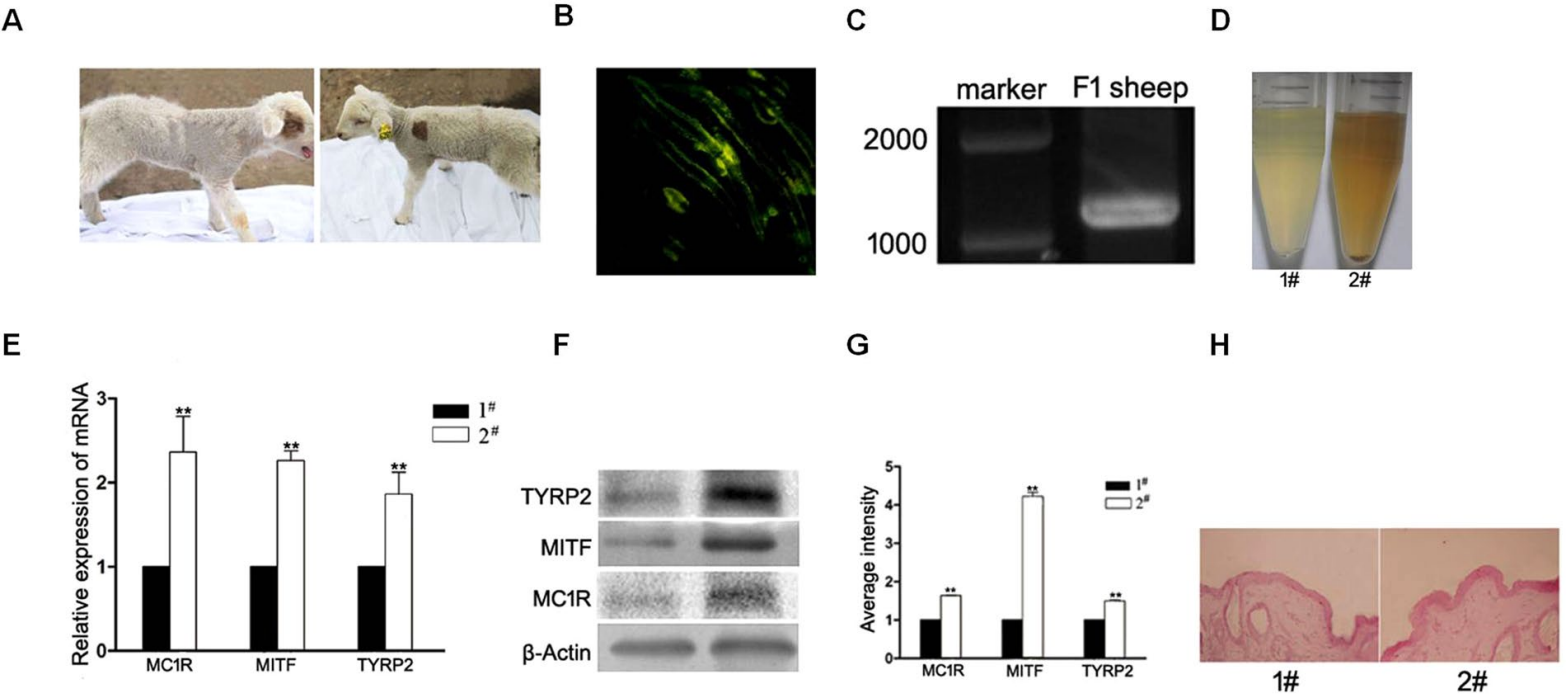
Phenotype of the transgenic lamb. (**A**) Brown patches on the right shoulder and around the left eye of the transgenic lamb. (**B**) Green fluorescence in the hair follicles of the transgenic lamb. (**C**) Target band from the total DNA of the transgenic lamb. (**D**) The melanin content from the hair of the white and brown patches of skin. (**E**) Melanogenic mRNA expression in the skin from the white and brown patches. (**F**) Melanogenic protein expression in the skin from the white and brown patches. #1 was from the white skin; #2 was from the brown patch. (**G**) The abundance of the TYRP2, MITF, and MC1R proteins was quantified using Image-Pro Plus software and normalized relative to the abundance of the β-actin protein. Bars represent the mean ± standard error (n = 3). ***P < 0.001. (**H**) H & E staining of the brown and white skin samples revealed the normal structure of the epidermis.

Due to the low efficiency of producing knockdown mice by microinjection of mouse zygotes, we chose to use testis germ cell transplantation to produce transgenic rams and offspring. The transplantation of 3 Dorset rams with testis germ cells transfected with a pLenti-Cdk5 construct resulted in all three producing transgenic sperm (Supplementary Figure 3). Mating of each male with 10 females produced a total of 23 lambs that were subsequently genotyped for the transgene by PCR. Of the 8 transgenic lambs, one (#6281) had brown patches on the right shoulder and around the left eye (Figs 8A) and 7 were white. Skin biopsies were collected from the brown patches on the right shoulders, and a white skin biopsy was collected from the opposite left shoulder. Frozen sections of both these skin biopsies showed green fluorescence, but the intensity was much stronger in the brown patch (Fig. 8B), indicating more copies of the Cdk5 expression. The target gene was amplified in the samples from the brown-patched lamb (Fig. 8C). The fluorescence was mainly observed in the epidermis and in some cells of the dermis. Although no differences were detected at the mRNA and protein levels for MC1R, MITF and TYRP2 in the brown-patched lamb, these genes were higher by 2.36-fold, 2.26-fold and 1.86-fold at the mRNA level, respectively, and 1.64-fold, 4.22-fold and 1.5-fold at the protein level, respectively, in the white skin compared to the brown skin (Fig. 8E and F). However, the melanin content was higher in the brown fleece than in the white fleece (Fig. 8D). H & E staining of the brown and white skin samples revealed a normal structure of the epidermis (Fig. 8H), indicating that the overexpression of Cdk5, unlike down-regulation, does not affect normal epidermal development.

## Discussion

An understanding of the mechanisms of skin pigmentation and coat-color determination not only provides a means to develop novel skin disease therapies but also has great potential for cosmetic applications in humans^9,36^. In addition, the ability to manipulate natural fleece color in fiber-producing species, such as alpacas and sheep, is in high demand in animal breeding programs. The avoidance of dyeing fleeces will provide environmentally friendly “green” products to the wool industry.

The coat color of mammals is dictated by the balance of two kinds of melanins, namely, eumelanin and pheomelanin, which are produced in melanocytes^9,22^. The anagen III-VI hair bulb is the only site of pigment production for the hair shaft and contains both strong melanogenic melanocytes and a small population of poorly differentiated pigment cells^37–39^. We previously demonstrated Cdk5 immunoreactivity in the upper hair bulb and outer root sheath in alpaca hair follicles and that *Cdk5* mRNA abundance was lower in alpacas with a white coat color than alpacas with a brown coat color^13,26^. The results of the present study using *Cdk5* overexpression and siRNA-mediated Cdk5 depletion in cultured melanocytes demonstrated a functional role for Cdk5 in the control of MC1R and TYR expression. Furthermore, partial depletion of Cdk5 resulted in a 70% reduction in total alkali-soluble melanin in the melanocytes. Cdk5 activity is induced by interactions with specific activator proteins, such as p35 and p39, under normal physiological conditions or by interactions with p25, which accumulates in the brains of Alzheimer’s patients^32,40^. Cdk5 coupled with the specific activator proteins has been shown to modulate the activity of a range of signaling pathways that modulate melanocyte functions, including the cyclic 3′5′-adenosine-monophosphate (cAMP)^41^, mitogen-activated protein kinase (MAPK)^42^ and Wnt signaling pathways^43^. This study identified p35 as an activator of Cdk5 in melanocytes.

Eumelanin is mostly synthesized in eumelanosomes, which have a characteristic ellipsoidal shape due to the accumulation of intraluminal fibrils (referred to as “the melanosome matrix”) and arise from the proteolytic processing of the premelanosomal protein (PMEL, also called Pmel17, gp100 or Silver in mice)^44,45^. PMEL fibrils have an amyloidogenic nature and share features with pathological amyloids^46^. The expression level of gp100 was reduced in the skin of the *Cdk5* knockdown mice. Amyloid fibrils are formed by various proteins in the pathologies of different neurodegenerative diseases such as Alzheimer’s and Parkinson’s diseases^47^. Because melanogenesis can affect the immune responses of the skin to the different chemotherapies and radiotherapies used to treat melanomas^48^, several drugs, including BRAF, KIT and MEK inhibitors, are currently being evaluated with promising results^49^. Taken together, with the activation by p35, Cdk5 regulates melanin production *via* the control of MC1R and TYR expression both at transcriptional and translational levels in melanocytes. Therefore, it is reasonable to speculate that Cdk5 can become a therapeutic target for melanoma and even for neurodegenerative pathologies such as Alzheimer’s and Parkinson’s diseases.

The finding that *in vivo* knockdown of *Cdk5* expression inhibits melanin production suggests that *Cdk5* is a promising target gene for the production of various coat colors in fleece- producing species. In addition, the localized distribution of melanin to the upper dermal papilla in the hair follicles of knockdown mice is another important factor for the altered coat color. The transportation of melanin along the dendrites of melanocytes is responsible, at least partially, for the lightened coat color from the original black background. Indeed, the reduced expression of MATP in the skin of the knockdown mice suggests that Cdk5 regulates MATP expression in the hair follicles. With the prediction using RNA-Seq, we have also shown that Cdk5 regulates the expression of a master gene of melanocyte development and melanogenesis, MITF, in the skin of transgenic mice. However, based on our current results, we are unable to confirm whether the functional role of Cdk5 on MC1R, TYRP1, TYRP2 and PAX3 expression occurs via regulation of MITF or whether Cdk5 can also directly influence the expression of these important downstream enzymes of melanogenesis. The downstream target of Cdk5 in this process may be PAX3, which has a binding site in the *MITF* promoter sequence that is known to be critical for transcriptional activation of *MITF* ^50^. Indeed, the expression for PAX3 in the skin of *Cdk5* knockdown mice was significantly reduced at both the mRNA and protein levels. This pathway is followed during melanogenesis. *Cdk5* knockdown induced a bright brown lightening of the new hair shafts that are produced during anagen, without inducing an ESR-detectable switch from eumelanogenesis to pheomelanogenesis. This phenomenon has also been reported as an effect of the oral zinc cations in C57BL/6 mice^51^. With the relative change in expression of TYRP1 and TYRP2, it is possible that eumelanin production in the hair of the *Cdk5* knockdown mice was the main factor in the coat color phenotype change, although MITF expression was also decreased. This conclusion supports the hypothesis that MITF is not the only transcription factor for melanogenesis^15^. However, the ratio of the relative expression of TYRP2 and TYRP1 cannot only affect the actual proportion of eumelanin/pheomelanin but also influence DHICA/DHI in eumelanin. Thus, hair color may be variable due to the changes in either the quantity or type of the main eumelanin monomer^7,14,51^. Moreover, we also found that expression of another receptor, melanocortin (MC) MC4R, was decreased in the skin of the *Cdk5* knockdown mice. It has been reported that MC4R in murine skin is tonically regulated by α-melanocyte stimulating hormone (α-MSH)^52^. Investigations of the regulation of Cdk5 on MC4R are underway.

A striking finding of this study was the observation of abnormal epidermal structure in the *Cdk5* knockdown mice. Using co-IP followed by mass spectrometry, we identified K10 as one of the substrates of CDK5. In the skin of the *Cdk5* knockdown mice, K10 was expressed significantly less than in the wild-type mice. It has been reported that the epidermis of adult Keratin 10 (K10)^−/−^ mice displayed hyperproliferation of basal keratinocytes and an increased cell size^21,53^. Furthermore, reduction of Cdk5 activity *via* an inhibitor (roscovitine) in HaCaT cells resulted in reduced adhesion of the cells to fibronectin, which was associated with reduced active states of β1 integrin^54^. In addition, the hair cycle determines skin thickness, which is highest during anagen VI and lowest during telogen in mice^39,55^. The results of the Western blot analysis showed that the expression level of transforming growth factor-βRII (TGF- βRII) was higher in the skin of the *Cdk5* knockdown mice, whereas the expression of interleukin (IL) -1RI (IL-1RI) was lower in comparison with the wild-type mice (Supplementary Figure 4). This indicates that there were more hair follicles at the anagen phase of the skin cycle of the *Cdk5* knockdown mice. Therefore, the thickness of the skin of *Cdk5* knockdown mice might also be caused by the growth phase of the hair cycle. Interestingly, the expression levels of Cdk5 and p35 in the lower half of the epidermis in psoriatic skin were reduced^54^. Collectively, these data demonstrate a requirement for Cdk5 in normal epidermal development to restrict hyperproliferation of the stratum spinosum and maintain the undifferentiated state of melanocytes. In addition, Cdk5 is also involved in the regulation of the hair cycle *via* the control of the TGF-βRII and IL-1RI expression levels. Therefore, we propose that reduced expression of Cdk5 in the epidermis might be involved in the pathogenesis of skin diseases such as psoriasis. Epidermal thickening could also potentially lead to the development of psoriasis or neurodermatitis (lichen simplex chronicus) in *Cdk5* knockdown animals if they were allowed to live longer, but further investigation is required to validate this hypothesis.

In contrast to *Cdk5* knockdown mice, lambs with skin-specific overexpression of Cdk5 did not show any abnormalities. However, unlike the knockdown mice where the coat color changed evenly over the body, the transgenic lamb (#6281) only had a brown patch on the body and around one eye where a higher level of green fluorescence was observed. Although all the cells in the transgenic sheep were supposed to contain the transgene, the expression levels of ectopic Cdk5 might have been different due to epigenetic factors. We suspect that a phenotypic change in the skin color can only be observed when the expression of Cdk5 exceeds certain limits. Similarly, a transgenic sheep carrying a transgene for the overexpression of the solute carrier family 7 member 11 (SLC7A11) gene, which is implicated in pheomelanin production, also exhibited brown/yellow patches in a white background^56^. These results may imply that coat-color determination in sheep is more complex than in rodents and is controlled by other novel genes. Indeed, several such genes have been identified in sheep skin, two of which were highly abundant and exclusively expressed in black sheep^57^. Furthermore, our previous study of the skin transcriptome profiles associated with coat color in sheep revealed that Cdk5 was not included in the top 30 highly expressed genes. It is puzzling for us to find that the mRNA and protein expression levels of MITF and TYRP2 were significantly reduced in brown versus white skin in the transgenic lambs since our current knowledge suggests that MITF and TYRP2 increase melanin production in melanocytes^7^. However, our studies and those of others have previously shown differential expression of Endothelin3 (Edn3) in white and black skin of Friesian sheep^58^ and color markings in domestic cats^59^, suggesting that paracrine expression of Edn3 might coordinate localized color differences. Furthermore, the melanin content in the brown fleece was higher than in the white fleece, implying potentially different pathways for regulating melanin production and coat-color determination in sheep. This also confirms that coat-color determination in sheep is more complex and controlled through autocrine, paracrine and endocrine mechanisms.

In summary, our results 1) demonstrate that Cdk5 is essential for normal epidermal development and melanogenesis, 2) highlight the involvement of Cdk5 in the pathogenesis of skin diseases and the clinical relevance of pharmaceutical correction of Cdk5, and 3) suggest *Cdk5* as a promising target gene for the generation of transgenic fleece-producing species.

## Methods

### Animals

The housing and care of animals and the collection of skin samples for use in the indicated experiments were approved by the Animal Ethics Committee of Shanxi Agricultural University (Taigu, Shanxi, PRC), and all experiments were performed in accordance with the relevant guidelines ([2010]088).

### Construction of the *Cdk5* expression vector

The complete alpaca *Cdk5* coding sequence (CDS) was amplified by RT-PCR (primers in Supplementary Table 2) and cloned into pVAX1 to produce pVAX1-Cdk5. Based on the CDS of *Cdk5*, 3 siRNAs (Supplementary Table 2) and one negative control (NC) siRNA (scrambled) were synthesized and transfected into alpaca melanocytes to screen for the more efficient siRNA to construct shRNA. The shRNA was then recombined with pENTR/U6 using the BLOCK-iT U6 RNAi Entry Vector Kit (Invitrogen, USA) to produce the pENTR/U6-Cdk5-shRNA construct. For transferring Cdk5 CDS into the sheep, the specific TYRP2 promoter was amplified by RT-PCR and cloned into a BamHI- and HindIII-digested pLenti vector to produce pLenti-TYRP2-Cdk5.

### Cdk5 overexpression and downexpression in alpaca melanocytes

A total of 1 × 10^5^ alpaca melanocytes were cultured for 24 h until they reached approximately 70% confluence. A total of 1.5 µg of the pVAX1-Cdk5 plasmid in serum-free medium was combined with 5 µl of the DNA Fectin transfection reagent (Tiangen Biotech, China) and incubated at room temperature for 20 min to form DNA-liposome complexes. Eight hundred microliters of serum-free medium plus the pVAX1-Cdk5 DNA/liposome complexes was added to the cultured melanocytes and incubated at 37 °C for 3 days for use.

For each siRNA transfection, 6 μl of siRNA was added to 100 μl of the transfection medium (Santa Cruz Biotechnology, USA) followed by addition of 6 μl of the siRNA transfection reagent (Santa Cruz Biotechnology). Melanocytes were seeded, cultured and transfected with the siRNAs and incubated for 6 h at 37 °C. The melanocytes were incubated for an additional 24 h for use.

### RNA isolation, cDNA synthesis and quantitative real-time PCR (qRT-PCR) analysis

Total RNA was isolated using TRIzol reagent (Invitrogen). One microgram of total RNA per sample was reverse transcribed to cDNA. qRT-PCR analysis of mRNA abundance for genes was conducted using SYBR Green (primers in Supplementary Table 2) and the comparative threshold cycle (Ct) method.

### H & E staining and Fontana-Masson staining

Skin tissue samples from the mice and sheep were embedded in paraffin, sectioned at 4 µm, and processed for either H & E or Fontana-Masson staining (Abcam, USA).

### Immunocytochemistry and immunohistochemistry

Cells for immunocytochemical analysis were rinsed briefly in PBS for 5 min, fixed in 4% paraformaldehyde for 20 min at 4 °C. Cells were then incubated with primary antibodies for 1 h at 37 °C at the following dilutions: polyclonal rabbit anti-Cdk5 antibody (1:200, Abcam), rabbit anti-MC1R (1:200, Santa Cruz Biotechnology) and rabbit anti-TYR (1:200, Santa Cruz Biotechnology). After incubation with appropriate secondary antibodies, images were obtained using a confocal microscope (Olympus FV1000, Japan).

The 4-µm paraffin skin sections were incubated in 3% hydrogen peroxide at room temperature for 30 min. After washing with PBS, the sections were blocked with 5% BSA at 37 °C for 30 min, followed by incubation with the anti-Cdk5 antibody (1:100, Abcam), anti-MATP antibody (1:100, Santa Cruz Biotechnology) or anti-K10 antibody (1:100, Abcam) at 4 °C for 14 h. Horseradish peroxidase (HRP)-conjugated goat anti-rabbit IgG was used as the secondary antibody, and DAB was used as the chromogenic substrate.

### Western blot analysis

Western blot analysis was performed as described^24^ with the following primary antibodies: polyclonal rabbit anti-TYR antibody (1:1000, Santa Cruz Biotechnology), polyclonal rabbit anti-β-actin antibody (1:800, Sigma, Germany), polyclonal rabbit anti-K10 antibody (1:1000, Abcam), rabbit anti-TYRP1 (1:1000, Abcam), mouse anti-MITF (1:1000, Thermo, USA), rabbit anti-PAX3 (1:200, Bioss), rabbit anti-MC1R (1:700, Abcam), polyclonal rabbit anti-Cdk5 antibody (1:200, Abcam), rabbit anti-gp100 (1:500, Abcam) and polyclonal rabbit anti-MC4R antibody (1:500, Abcam), rabbit anti-IL1R (1:500, Abcam), rabbit anti-NASM antibody (1:500, Abcam) and rabbit TGFβIIR (1:500, Abcam). Immunoblots were scanned on a ChemiDOC^TM^ XRS + imager (Bio-Rad, USA), and protein levels were quantified using Image-Pro Plus software (Olympus, Japan).

### Co-immunoprecipitation assays

Melanocytes were lysed in lysis buffer, and anti-Cdk5 primary antibody was added to 200 μl of the cell lysate followed by incubation overnight at 4 °C. Twenty microliters of protein G agarose beads (50% bead slurry) was then added to the mixture and incubated for 2 h at 4 °C and then centrifuged at 3,000 g for 3 min to get the pellets, which were resuspended, heated at 95 °C for 5 min and loaded onto an SDS-PAGE gel (12–15% gradient). Western blot was performed using an anti-p35 antibody, anti-K10 antibody or anti-Cdk5 antibody. Reciprocal co-IP was performed using an anti-p35 antibody or anti-K10 antibody to co-precipitate Cdk5.

### Identification of Cdk5-interacting proteins by mass spectrometry

Total proteins from mouse melanocytes and 30 µl of protein-A/G agar beads were mixed. The mixture was placed at 4 °C for 1 h and then centrifuged at 3,000 g for 3 min. The supernatant was obtained and mixed with an anti-Cdk5 antibody. After incubation overnight, 50 µl of the protein-A/G agar beads were added, and the mixture was incubated for 2 h followed by centrifugation at 3,000 g for 3 min. The precipitated proteins were then analyzed by LC-ESI-Q-TOF mass spectrometry. The International Protein Index (IPI) protein database (http://www.ebi.ac.uk/IPI) was used for protein identification.

### Generation of *Cdk5* knockdown mice

After **t**he pENTR/U6-Cdk5-shRNA construct was linearized by restriction digestion, gel purified, and resuspended in the injection buffer (Sigma) at a concentration of 3 ng/µl, it was microinjected into the male pronuclei of the zygotes of C57BL/6 J mice. Injected embryos were transferred into the oviducts of the pseudopregnant recipient females. The transgenic lines were maintained by backcrossing with C57BL/6 J mice, which were used as the control with a stable genetic background. Genotyping was performed by PCR using tail DNA as the templates.

### Production of transgenic sheep with skin tissue-specific Cdk5 overexpression

Three healthy 2-year-old Dorset rams were selected randomly and treated with 12 Gy radiation. Six weeks following the irradiation, one healthy 15-month-old Dorset ram was castrated, and the testicular tissues were used to prepare testis germ cells. After single cell suspensions were prepared by enzyme digestion, germ cells were exposed to AAV vectors carrying the eGFP reporter gene under the control of the pLenti-Tyrp2-Cdk5 plasmid at 2,000–12,000 gc/cell and centrifuged at 500 g for 30 min. The transfected cells (50–60 × 10^6^) were transferred to T250 flacks and cultured at 37 °C in 5% CO_2_ overnight. Within 2 h, 150–200 × 10^6^ cells in 5 ml DMEM/12 medium with 5% fetal bovine serum were transplanted into the rete testis of each testicle of the previously irradiated rams. After 13 weeks, the semen was collected from each recipient and genotyped for the transgene. Each of the Cdk5-positive rams was mated to 10 ewes. After the lambs were born, DNA was isolated from the skin biopsies, and the transgene was amplified by PCR using specific primers (Supplementary Table 2).

### RNA-Seq analysis

Total RNA from the skin of the *Cdk5* knockdown and wild-type mice (n = 3) was isolated for deep sequencing. The short cDNA fragments were produced and purified using the QiaQuick PCR extraction kit (Qiagen, USA), and cDNA libraries were constructed after the fragment ends were repaired and tailed followed by ligation to sequencing adapters. Sequencing of the libraries was performed using the Illumina HiSeq™ 2000. A rigorous algorithm (http://www.genetics.cn) was used to identify differentially expressed genes in the skin between the *Cdk5* knockdown and wild-type mice.

### Determination of melanins in cultured melanocytes and hair

Melanocytes were trypsinized, centrifuged at 10,000 g for 10 min and solubilized in 1 M NaOH. Hair was washed in mild detergent and then hydrolyzed in 1 M NaOH at 85 °C for 4 h. The concentrations of the alkali-soluble melanins (pheomelanins and some eumelanins) in the cell lysates or hair were calculated from spectrophotometric absorbance measurements at 475 nm, using a standard curve that was prepared with serial dilutions of a Sepia melanin suspension (1 mg/ml). Forty-milligrams of mice hair were collected, washed in xylene and dried at room temperature. Hair samples were placed into the glass tubes, and ESR signals were recorded using an ESR spectrometer^60^.

### Statistical analysis

Differences in the mRNA, protein and melanin production abundances between the treatment groups (n = 3 replicates) were determined by analysis of variance using SPSS 11.5 software.

## Electronic supplementary material

Supplemental Info

## References

[1] Chuong CM (2002). What is the ‘true’ function of skin?. Exp. Dermatol..

[2] Tobin DJ (2006). Biochemistry of human skin-our brain on the outside. Chem. Soc. Rev..

[3] Liu J, Fukunaga-Kalabis M, Li L, Herlyn M (2014). Developmental pathways activated in melanocytes and melanoma. Arch. Biochem. Biophys..

[4] Plonka PM (2009). Electron paramagnetic resonance as a unique tool for skin and hair research. Exp. Dermatol..

[5] Wood JM (1999). What’s the use of generating melanin?. Exp. Dermatol..

[6] Brozyna AA, Jozwicki W, Roszkowski K, Filipiak J, Slominski AT (2016). Melanin content in melanoma metastases affects the outcome of radiotherapy. Oncotarget.

[7] Slominski A, Tobin DJ, Shibahara S, Wortsman J (2004). Melanin pigmentation in mammalian skin and its hormonal regulation. Physiol. Rev..

[8] Slominski A, Zmijewski MA, Pawelek J (2012). L-tyrosine and L-dihydroxyphenylalanine as hormone-like regulators of melanocyte functions. Pigment Cell Melanoma Res..

[9] Yamaguchi Y, Brenner M, Hearing VJ (2007). The regulation of skin pigmentation. J. Biol. Chem..

[10] Slominski A, Wortsman J, Luger T, Paus R, Solomon S (2000). Corticotropin releasing hormone and proopiomelanocortin involvement in the cutaneous response to stress. Physiol. Rev..

[11] Fitzpatrick TB (1965). Mammalian melanin biosynthesis. Trans. St Johns Hosp. Dermatol. Soc..

[12] Lin YP, Hsu FL, Chen CS, Chern JW, Lee MH (2007). Constituents from the Formosan apple reduce tyrosinase activity in human epidermal melanocytes. Phytochemistry.

[13] Hornyak TJ, Hayes DJ, Chiu LY, Ziff EB (2001). Transcription factors in melanocyte development: distinct roles for Pax-3 and Mitf. Mech. Dev..

[14] Schallreuter KU, Kothari S, Chavan B, Spencer JD (2008). Regulation of melanogenesis-controversies and new concepts. Exp. Dermatol..

[15] Vachtenheim J, Borovansky J (2010). “Transcription physiology” of pigment formation in melanocytes: central role of MITF. Exp. Dermatol..

[16] Bentley NJ, Eisen T, Goding CR (1994). Melanocyte-specific expression of the human tyrosinase promoter: activation by the microphthalmia gene product and role of the initiator. Mol. Cell Biol..

[17] Hemesath TJ (1994). Microphthalmia, a critical factor in melanocyte development, defines a discrete transcription factor family. Genes Dev..

[18] Yasumoto K, Yokoyama K, Shibata K, Tomita Y, Shibahara S (1994). Microphthalmia-associated transcription factor as a regulator for melanocyte-specific transcription of the human tyrosinase gene. Mol. Cell Biol..

[19] Zhu Z (2010). MicroRNA-25 functions in regulation of pigmentation by targeting the transcription factor MITF in alpaca (*Lama pacos*) skin melanocytes. Domest. Anim. Endocrinol..

[20] Medic S, Ziman M (2009). PAX3 across the spectrum: from melanoblast to melanoma. Crit. Rev. Biochem. Mol. Biol..

[21] Lang D, Epstein JA (2003). Sox10 and Pax3 physically interact to mediate activation of a conserved c-RET enhancer. Hum. Mol. Genet..

[22] Lang D (2005). Pax3 functions at a nodal point in melanocyte stem cell differentiation. Nature.

[23] Tian X (2012). Identification and characterization of microRNAs in white and brown alpaca skin. BMC Genomics.

[24] Dong C (2012). Coat color determination by miR-137 mediated down-regulation of microphthalmia-associated transcription factor in a mouse model. RNA.

[25] Fan R (2011). Gene expression profile in white alpaca (*Vicugna pacos*) skin. Animal.

[26] Liu J (2010). Expression of cyclin dependent kinase 5 in alpaca skin of different hair colors. Acta Veterinaria et Zootechnica Sinica.

[27] Dhariwala FA, Rajadhyaksha MS (2008). An unusual member of the Cdk family: Cdk5. Cell. Mol. Neurobiol..

[28] Donoghue PC, Graham A, Kelsh RN (2008). The origin and evolution of the neural crest. Bioessays.

[29] Hou L, Pavan WJ (2008). Transcriptional and signaling regulation in neural crest stem cell-derived melanocyte development: do all roads lead to Mitf?. Cell Res..

[30] Tsai LH, Takahashi T, Caviness VS, Harlow E (1993). Activity and expression pattern of cyclin-dependent kinase 5 in the embryonic mouse nervous system. Development.

[31] Lew J, Beaudette K, Litwin CM, Wang JH (1992). Purification and characterization of a novel proline-directed protein kinase from bovine brain. J. Biol. Chem..

[32] Philpott KL, McCarthy MJ, Klippel A, Rubin LL (1997). Activated phosphatidylinositol 3-kinase and Akt kinase promote survival of superior cervical neurons. J. Cell Biol..

[33] Liebl J (2015). Cdk5 controls lymphatic vessel development and function by phosphorylation of Foxc2. Nat. Commun..

[34] Husson H (2016). Reduction of ciliary length through pharmacologic or genetic inhibition of CDK5 attenuates polycystic kidney disease in a model of nephronophthisis. Hum. Mol. Genet..

[35] Lee KY (2004). Cdk5/p35 expression in the mouse ovary. Mol. Cells.

[36] Kornhauser A (2009). The effects of topically applied glycolic acid and salicylic acid on ultraviolet radiation-induced erythema, DNA damage and sunburn cell formation in human skin. J. Dermatol. Sci..

[37] Slominski A, Paus R (1993). Melanogenesis is coupled to murine anagen: toward new concepts for the role of melanocytes and the regulation of melanogenesis in hair growth. J. Invest. Dermatol..

[38] Tobin DJ, Bystryn JC (1996). Different populations of melanocytes are present in hair follicles and epidermis. Pigment Cell Res..

[39] Muller-Rover S (2001). A comprehensive guide for the accurate classification of murine hair follicles in distinct hair cycle stages. J. Invest. Dermatol..

[40] Lopes JP, Agostinho P (2011). Cdk5: multitasking between physiological and pathological conditions. Prog. Neurobiol..

[41] Guan JS (2011). Cdk5 is required for memory function and hippocampal plasticity via the cAMP signaling pathway. PLoS One.

[42] Hisanaga S, Endo R (2010). Regulation and role of cyclin-dependent kinase activity in neuronal survival and death. J. Neurochem..

[43] Li Q (2010). Characterization of a novel human CDK5 splicing variant that inhibits Wnt/beta-catenin signaling. Mol. Biol. Rep..

[44] Bissig, C., Rochin, L. & van Niel, G. PMEL amyloid fibril formation: the bright steps of pigmentation. *Int. J. Mol. Sci*. **17** (2016). 10.3390/ijms17091438 PMC5037717 27589732

[45] Zhou BK (1994). Identification of a melanosomal matrix protein encoded by the murine si (silver) locus using “organelle scanning”. Proc. Natl. Acad. Sci. USA.

[46] Fowler DM (2006). Functional amyloid formation within mammalian tissue. PLoS Biol..

[47] Knowles TP, Vendruscolo M, Dobson CM (2014). The amyloid state and its association with protein misfolding diseases. Nat. Rev. Mol. Cell Biol..

[48] Brozyna AA, Jozwicki W, Carlson JA, Slominski AT (2013). Melanogenesis affects overall and disease-free survival in patients with stage III and IV melanoma. Hum. Pathol..

[49] Spagnolo F, Queirolo P (2012). Upcoming strategies for the treatment of metastatic melanoma. Arch. Dermatol. Res..

[50] D’Mello SA, Finlay GJ, Baguley BC, Askarian-Amiri ME (2016). Signaling pathways in melanogenesis. Int. J. Mol. Sci..

[51] Plonka PM, Handjiski B, Michalczyk D, Popik M, Paus R (2006). Oral zinc sulphate causes murine hair hypopigmentation and is a potent inhibitor of eumelanogenesis *in vivo*. Br. J. Dermatol..

[52] Kaelin CB (2008). New ligands for melanocortin receptors. Int. J. Obes..

[53] Reichelt J, Magin TM (2002). Hyperproliferation, induction of c-Myc and 14-3-3sigma, but no cell fragility in keratin-10-null mice. J. Cell Sci..

[54] Nakano N (2005). CDK5 regulates cell-cell and cell-matrix adhesion in human keratinocytes. Br. J. Dermatol..

[55] Michalczyk D, Popik M, Salwinski A, Plonka PM (2009). Extradermal melanin transfer? Lack of macroscopic spleen melanization in old C57BL/6 mice with de-synchronized hair cycle. Acta Biochim. Pol..

[56] He X, Li H, Zhou Z, Zhao Z, Li W (2012). Production of brown/yellow patches in the SLC7A11 transgenic sheep via testicular injection of transgene. J. Genet. Genomics.

[57] Fan R (2013). Skin transcriptome profiles associated with coat color in sheep. BMC Genomics.

[58] Yang S-S (2015). The different expression of endothelin 3 in sheep skins. Acta Veterinaria et Zootechnica Sinica.

[59] Kaelin CB (2012). Specifying and sustaining pigmentation patterns in domestic and wild cats. Science.

[60] Plonka PM, Michalczyk D, Popik M, Handjiski B, Paus R (2008). Electron paramagnetic resonance (EPR) spectroscopy for investigating murine telogen skin after spontaneous or depilation-induced hair growth. J. Dermatol. Sci..

