# Supplementary material for: Functional Role of Cyclin-Dependent Kinase 5 in the Regulation of Melanogenesis and Epidermal Structure
Source: Sci Rep. 2017 Oct 23;7:13783. doi: 10.1038/s41598-017-12567-1 (PMC5653820; doi:10.1038/s41598-017-12567-1)
Supplement: Supplementary file 1 — Supplemental Info [file 41598_2017_12567_MOESM1_ESM.pdf]

Functional Role of Cyclin-Dependent Kinase 5 in the Regulation of Melanogenesis and Epidermal Structure

Changsheng Dong[1*], Shanshan Yang[1], Ruiwen Fan[1*], Kaiyuan Ji[1], Junzhen Zhang[1], Xuexian Liu[1], Shuaipeng Hu[1], Jianshan Xie[1], Yu Liu[1], Wenjun Gao[1], Haidong Wang[1], Jianbo Yao[1,2], George W Smith[1,3], and Muren Herrid[1,4]

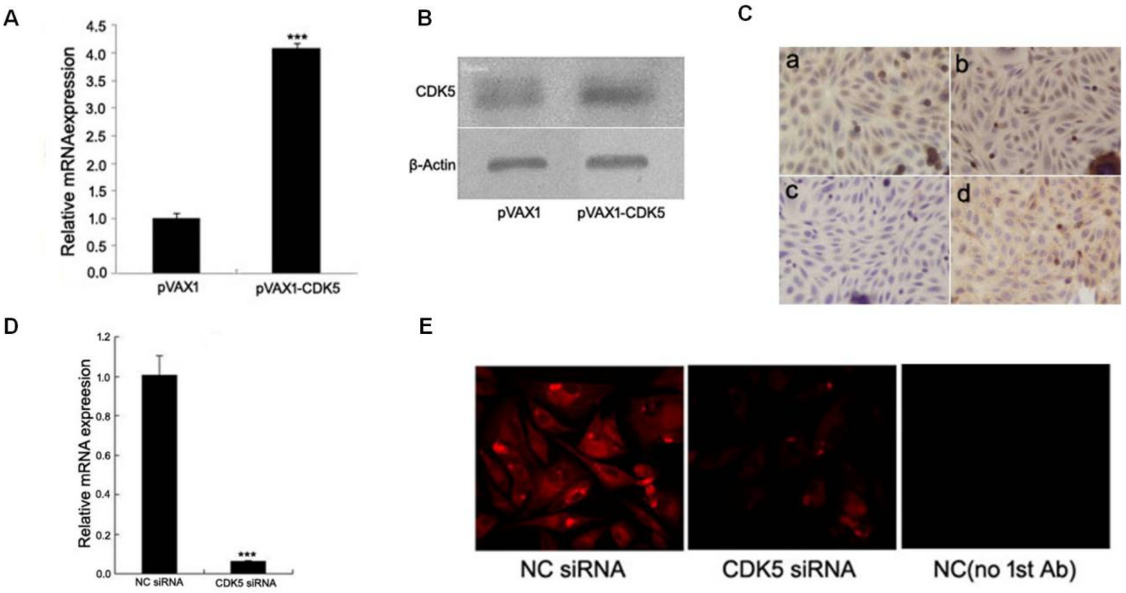

Supplementary Fig. 1. Analysis of CDK5 protein expression in melanocytes transfected by pVAX1-Cdk5 and siRNA-Cdk5. (A) mRNA expression of Cdk5 in melanocytes transfected by pVAX1-Cdk5 (B) protein expression of Cdk5 in melanocytes transfected by pVAX1-Cdk5. (C) Analysis of Cdk5 protein expression in negative control siRNA. **a**: Cdk5 siRNA-531. **b**: siRNA-171. **c**: siRNA-215. **d**: NC. (D) Effect of Cdk5 siRNA-215 on Cdk5 mRNA in cultured melanocytes. (E) Effect of Cdk5 siRNA-215 on Cdk5 protein abundance in cultured melanocytes transfected melanocytes. Experiment was replicated three times.

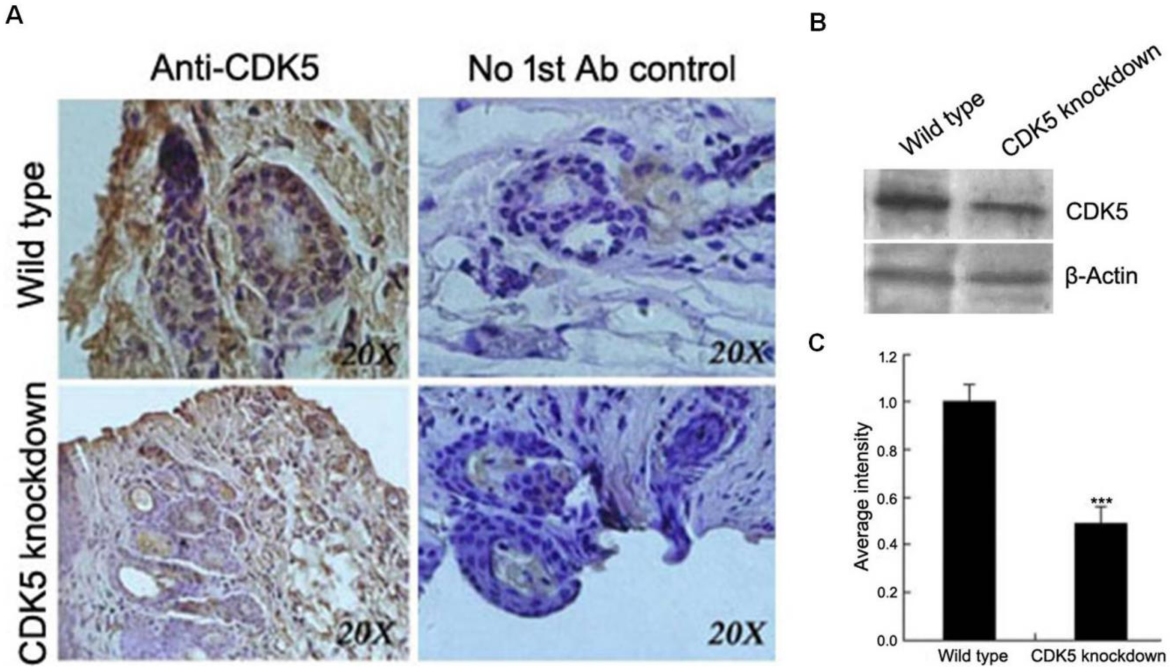

Supplementary Fig. 2. Analysis of Cdk5 protein expression in the skin of Cdk5 knockdown mice. (A) Immunohistochemical analysis of Cdk5 protein expression in Cdk5 knockdown mice skin. (B) Western blot analysis of Cdk5 protein expression in the skin of Cdk5 knockdown mice compared with wild-type mice. (C) Abundance of Cdk5 protein was quantified using the Image-Pro Plus software and normalized relative to abundance of β-actin protein. Bars represent mean ± standard error (n = 3). *** P<0.001.

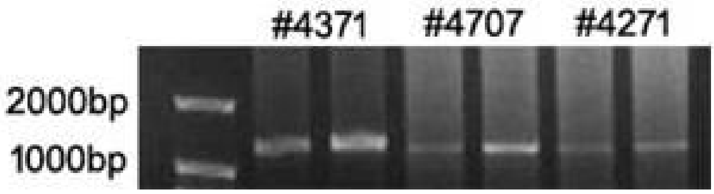

Supplementary Fig. 3. Identification of Cdk5 overexpression ram. Detection of transgene in semen from three rams by RT-PCR.

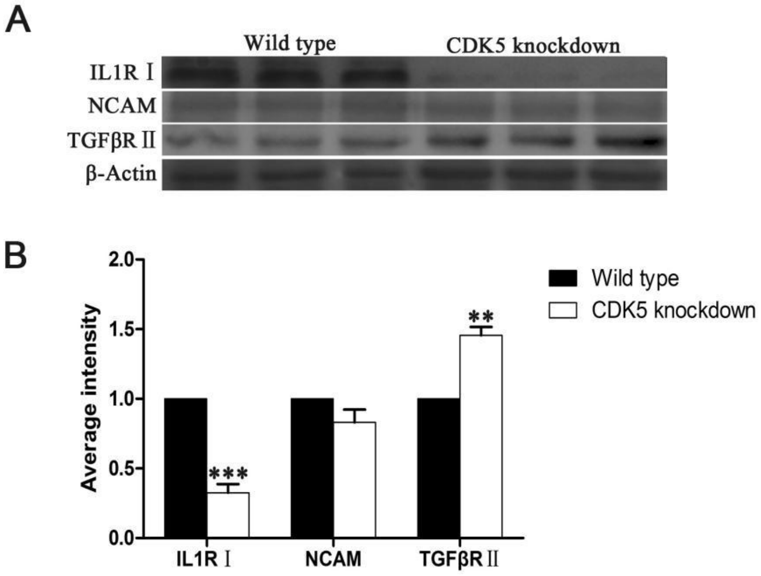

Supplementary Figure 4. Analysis of protein expression of molecular markers for hair cycle stages in the skin of Cdk5 knockdown mice. (A) Western blotting analysis of TGF-βRII, NCAM, IL-1R protein expression using antibodies against TGF-βRII (rabbit anti-TGF-βRII, 1:500, Abcam), NCAM (rabbit anti-NCAM, 1:500, Abcam), and IL-1R (rabbit anti-IL-1R, 1:500, Abcam) in Cdk5 knockdown mice skins. (B) Abundance of TGF-βRII, NCAM, IL-1R protein was quantified using the Image-Pro Plus software and normalized relative to abundance of β-actin. Bars represent mean ± standard error (n=3). ** $P < 0.01$, *** $P < 0.01$

Supplementary Table 1. Down-regulated coat color genes in Cdk5 knockdown skin.

Gene Name	Fold Changes	Classification	Function
**Melanocortin 1 receptor (MC1R)**	2.89	Seven transmembrane G protein-coupled receptors	Regulating melanogenesis, cell proliferation and survival
**Tyrosinase-related protein 1 (TYRP1*)***	13.68	Components of melanosomes and their precursors	Melanosomal protein
**Tyrosinase-related protein 2 (TYRP2)**	11.63	Pigmentation enzymes	Melanosomal protein
**Microphthalmia-associated transcription factor (MITF)**	15.38	Transcriptional regulator	Regulating cell differentiation, proliferation and survival
**Paired box 3 (PAX3)**	13.05	Transcriptional regulator	Regulating cell proliferation, migration and activating MITF expression

Supplementary Table 2. Identification of Cdk5 overexpression ram

Gene	Primers (5′→3′)	Application
**CDK5**	F: TTGGCTGATTTTGGCTTG<br>R: TCGTTGCCAGGAAAGAGAG	Real time PCR
**TYR**	F: TGCCATCTTTGATGAGTGG<br>R: CTGGAGGGAAGAAAGGAAC	Real time PCR
**MC1R**	F: TCTATGCACTGCGCTACCAC<br>R: GACATATAGCACCGCCATGA	Real time PCR
**18S rRNA**	F: GAAGGGCACCACCAGGAGT<br>R: CAGACAAATCACTCCACCAA	Real time PCR
**siRNA-215**	GCGACAAGAAGCUGACUUUdTdT<br>AAAGUCAGCUUCUUGUCGCdTdT	RNAi
**siRNA-531**	GCUGUACUCCACGUCCAUUdTdT<br>AAUGGACGUGGAGUACAGCdTdT	RNAi
**siRNA-171**	GCUGAAGCACAAGAACAUUdTdT<br>AAUGUUCUUGUGCUUCAGdTdT	RNAi
**CDK5**	F: ATGCAGAAATACGAGAAACTG<br>R: CTAGGGAGGGCAGAAGTCGGA	RT-PCR
**CDK5**	F:GGGCTAGAACTAAACCCAAAG<br>R: CCAAAAGACGGCAATATGGTG	Transgenic sheep test
**CDK5**	F:TGTGGAAAGGACGAAACACC<br>R:CAGCTGGATGGCAAATAATG	Transgenic mice test
**CDK5**	F:GAGCCATATTCAACGGGAAA<br>R:GATGGTCGGAAGAGGCATAA	Transgenic mice test

F (forward primer), R (reverse primer).

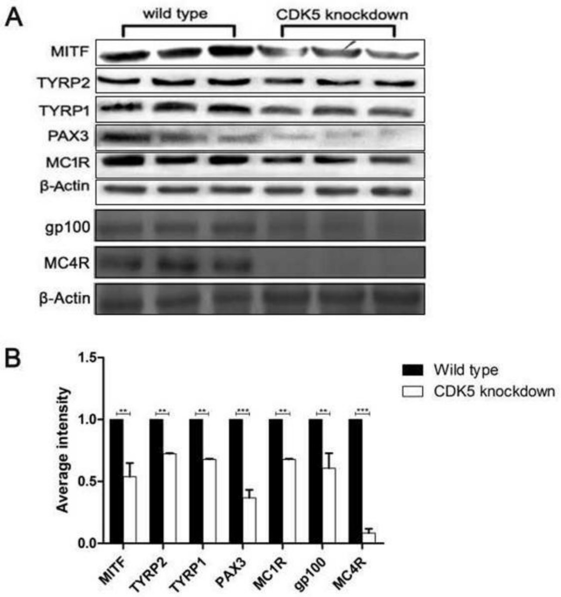

Fig.5

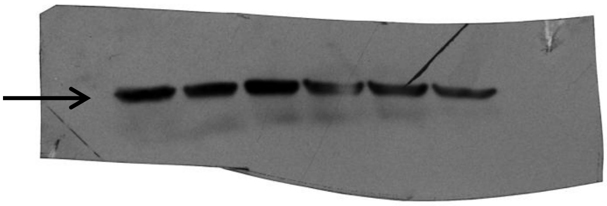

MITF

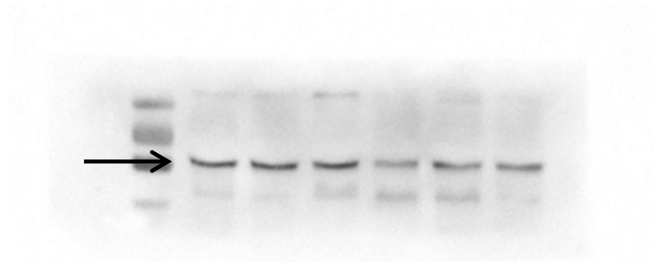

TYRP2

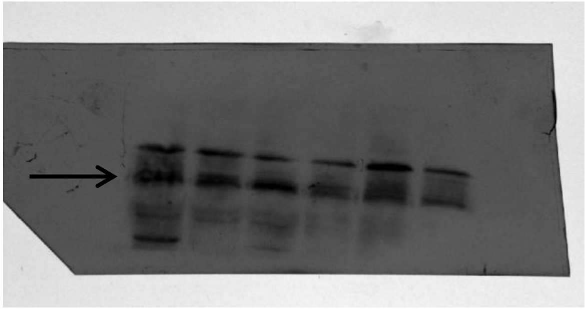

TYRP1

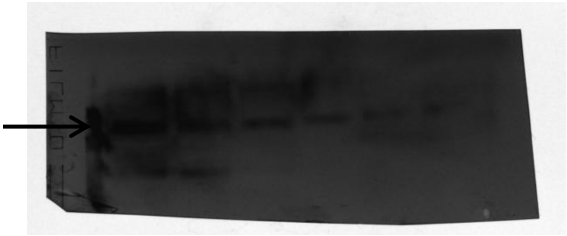

PAX3

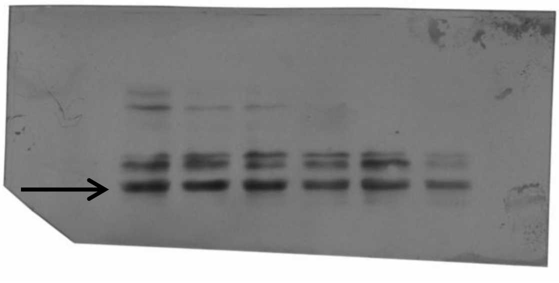

MC1R

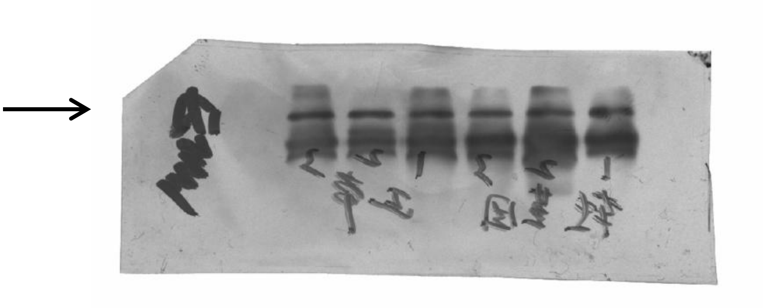

β-Actin

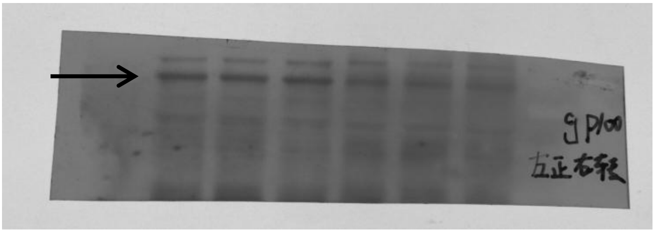

gp100

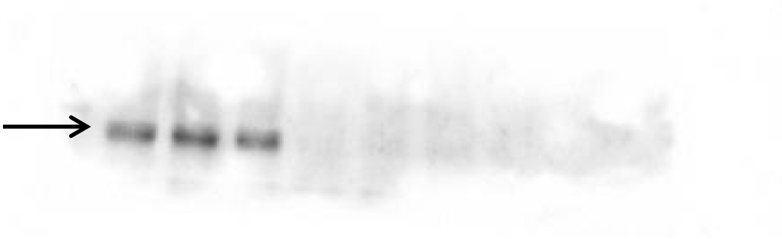

MC4R

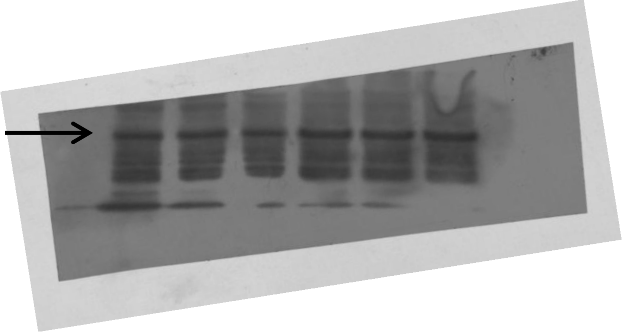

β-Actin
